# Hysterotomy for Retained Placenta in a Septate Uterus: A Case Report

**DOI:** 10.1155/2012/594140

**Published:** 2012-06-07

**Authors:** Daniel Lee, Joseph Johnson

**Affiliations:** Department of Obestrics and Gynecology, Oklahoma State University, 717 S. Houston Avenue, Suite 200, Tulsa, OK 74127, USA

## Abstract

Retained placenta is a common complication of the third stage of labor. Most literature has focused on management of a trapped placenta or placenta accreta. The most common source of a trapped placenta is from a partial closure of the cervix and/or a contracted lower uterine segment. We present an unusual case of a retained placenta trapped in a septate uterus. The management included unsuccessful conservative measures that resulted in delivery of the placenta by laparotomy with hysterotomy.

## 1. Introduction

A retained placenta prevents normal uterine contraction, which can lead to excessive hemorrhage. The management to deliver the placenta and prevent a large hemorrhage depends on the etiology of the retained placenta. Commonly, the cause is from a partial closure of the cervix and/or a contracted lower uterine segment [1]. A septate uterus is a uterine anomaly associated with spontaneous abortion and preterm delivery [2]. We present a rare case of a trapped placenta in a known septate uterus after a preterm delivery at 35 weeks. We were unable to deliver the placenta after standard conservative measures and thus proceeded to surgical management.

## 2. Case Report

**Figure 1:**
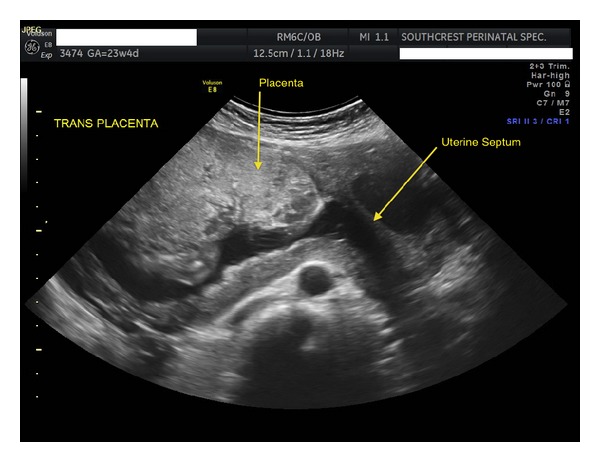
23-week transverse view of septate uterus.

A 34 year-old female, gravida 1 para 0, at 35 weeks was admitted to the hospital in preterm labor. She had been followed throughout her pregnancy by a perinatologist with concerns of preterm delivery secondary to a prominent septate uterus (Figure 1).

She was started on penicillin for GBS prophylaxis. During the second stage of labor she had nonreassuring fetal heart tones. The 5-pound-0-ounce infant was delivered by outlet forceps-assisted vaginal delivery with apgars of 4 at 1 minute and 7 at 5 minutes. A second degree midline perineal laceration was repaired in standard fashion. This was done after 10 minutes of waiting on placenta delivery and to prevent any excess bleeding from the laceration site.

After forty minutes of cord traction, uterine massage, IV pitocin (20 units in 500 mL normal saline at 125 mL/hr), and attempted manual extraction with IV stadol 2 mg for pain, we were unable to deliver the placenta. She was taken to the operating room for manual extraction after spinal anesthesia was placed. Multiple attempts were made at manual removal under direct ultrasound visualization. We were unable to reach the placenta as the septum and the contraction of the lower uterine segment blocked our ability to reach it at its fundal location in the right horn. A sharp uterine currettage was performed, again without success, as the curette barely reached the edge of the placenta. It was clear at this point that the placenta was trapped and not accessible for delivery without general anesthesia.

Consent was obtained after discussion with the patient and her husband to proceed with general anesthesia and one last attempt for manual removal and possible curettage; however, if unsuccessful, we would proceed with a laparotomy and hysterotomy with possible hysterectomy if there was evidence of myometrial invasion of the placenta. We understood their desire for uterine preservation for future fertility. After general anesthesia was obtained, there was appropriate uterine relaxation but we were unable to remove the placenta via manual attempt and currettage. The placenta remained trapped in the right uterine horn, and the septum continued to block appropriate access. A low transverse uterine incision was then made and we were still unable to reach the placenta that was trapped in the right uterine horn. Thus, a reversed “J-” shaped uterine incision was extended up to the fundus on the right side. The placenta was then identified and manually removed. The placenta was removed entirely intact, and there was no evidence of abnormal invasive placentation. The uterus was closed in a three-layer closure. The estimated blood loss was 1,000 mL. Her preoperative hemoglobin was 13.6 and postoperative day 1 hemoglobin was 9.8. She did not require blood product transfusion. The remainder of her postpartum course was unremarkable, and she was discharged home in stable condition on postoperative day 4. Upon discharge, she was appropriately counseled on the need for a repeat cesarean delivery given her uterine incision. We also recommended her to have an outpatient consultation by a reproductive infertility specialist regarding a metroplasty to decrease her risk of preterm delivery and recurrent retained placenta in future pregnancies.

## 3. Discussion

The incidence of retained placenta varies greatly around the world, affecting between 0.1 and 3.3% of vaginal deliveries depending on the population studied [1]. Maternal mortality is as high as 9% in the developing world [3]. The cause of death is usually hemorrhage. Obviously appropriate medical facilities allowing for surgical management if needed can have major implications for the reduction of maternal mortality.

There is no consensus worldwide as to the length of the third stage after which a placenta should be termed “retained” and intervention initiated. Intrapartum guidelines produced for the National Institute of Health and Clinical Excellence (NICE) suggest intervention when the placenta has been retained for 30 minutes after birth [4], while the World Health Organization (WHO) recommends waiting for 60 minutes [5]. At our institution, retained placenta is defined after 30 minutes. In our case, manual extraction was attempted at 40 minutes into stage 3. However, the patient did not tolerate the manual extraction attempt after given IV stadol.

At our institution “active management” of third stage of labor is practiced, specifically administration of pitocin prior to placenta delivery. Although the data published over the last ten years shows mixed results, there appears to be a possible decrease in postpartum hemorrhage, total blood loss, and length of time of the third stage [6, 7]. There does not appear to be an increased risk of a trapped placenta with pitocin administered prior to placenta delivery [7].

At this point in the management other options include umbilical vein prostaglandin injection or facilitating uterine relaxation with nitroglycerin or general anesthesia. We have not implemented the prostaglandin injection process at our institution, but there are small randomized trials that have shown some success. These studies look at umbilical vein injection of prostaglandin F2*α* (20 mg in 20 mL of normal saline) and dissolved misoprostol (800 mcg in 30 mL of normal saline) [8, 9]. Umbilical vein injection with oxytocin does not appear to be effective. A large, multicenter, placebo-controlled, and randomized trial in which 577 women with retained placenta were given high-dose oxytocin (50 units in 30 mL saline) or an equivalent volume of saline showed no differences between groups in rate of manual placental removal or frequency of adverse events [10].

Nitroglycerin has also been described in the literature when there is partial closure of the cervix and/or a contracted lower uterine segment preventing placenta delivery. It can be administered to relax the uterus and facilitate delivery [11, 12]. We did not use nitroglycerin; however, it has been our experience that general anesthesia often facilitates delivery of the trapped placenta that has not responded to more conservative measures. However in this case we did not expect that is was going to work because the placenta clearly appeared anatomically trapped by the septum more than the lower uterine segment.

There are a number of pathologies that lead to a retained placenta [1]. Some placentas are trapped behind a closed cervix or a tightened down lower uterine segment (trapped placenta), some are adherent to the uterine wall but easily separate manually (placenta adherens), and others are invading the myometrium (placenta accreta). In our case the placenta was trapped in the right fundal horn of a septate uterus. We were unable to reach the placenta even after spinal and general anesthesia. This case is very similar to previously documented cases of “angular pregnancies.” An angular pregnancy is a rare condition in which the embryo implants in the lateral angle of the uterine cavity, just medial to the uterotubal junction [13]. Unlike interstitial pregnancies, these pregnancies have a much favorable outcome. A PubMed search shows only two cases of angular pregnancy complicated by retained placenta requiring hysterotomy for placenta delivery [14, 15].

We are unaware of any documented cases describing management of a trapped placenta in a septate uterus. Fortunately we had the ability to perform a hysterotomy to remove the placenta and preserve her fertility. If the placenta had invaded into her myometrium, she, very well likely, would have needed a hysterectomy.
